# Disparities in Tobacco Use, Binge Drinking, and Injection Drug Use Among Sexual and Gender Minoritized People Recruited Online in San Francisco, California: Survey Results

**DOI:** 10.2196/81982

**Published:** 2025-11-17

**Authors:** Sean Arayasirikul, Jarett Maycott, Arianna Contestable

**Affiliations:** 1 The Legacy Center Joe C Wen School of Population & Public Health University of California, Irvine Irvine, CA United States; 2 San Francisco Department of Public Health San Francisco, CA United States

**Keywords:** substance use, health disparities, sexual and gender minorities, community health, social media

## Abstract

This research letter describes substance use disparities among online, help-seeking, sexual and gender minoritized people in San Francisco. Our findings emphasize the importance of strengthening public health practice by leveraging digital and online methods to reach communities.

## Introduction

The complexity of substance use as a public health issue is multifaceted. Compared to the general population, people who use substances are more likely to have hospital and emergency department visits, often presenting with an immediate need for treatment (eg, overdose) or being a source of harm to others (eg, driving under the influence) [1]. Research has identified people who use substances as a population that utilizes greater health care services at higher costs [2]. In response to this strain on the public health care system, specialized services such as sobering centers and supervised injection facilities have become critical harm reduction assets [3,4]. While the public health infrastructure and tools to prevent substance use disorder has improved to address the opioid epidemic, people who use substances experience substance use stigma as a barrier to accessing substance use services [5]. Substance use impacts diverse marginalized communities, especially sexual and gender minoritized (SGM) communities. SGM individuals are more likely to use substances compared to cisgender heterosexual individuals [6,7].

## Methods

### Study Design

We conducted a cross-sectional analysis of 409 adult men who have sex with men and transgender women and gender expansive (TGE) in San Francisco. Participants interested in receiving digital navigation services for substance use and related health topics (eg, mental health, HIV) were recruited using advertisements on Facebook, Instagram, and Grindr in 2022-2024. We analyzed the sociodemographic characteristics. Race/ethnicity was recoded to a dichotomous variable with Black, indigenous, and people of color (BIPOC) people=1 and White=0. We assessed participants’ recent substance use behaviors by asking how many days in the last 30 days, if any, did they use the following substances [8]: tobacco, vaping products, binge drinking, marijuana, prescription opioids not prescribed to you (eg, pain relievers), nonprescription opioids (eg, heroin, fentanyl), other prescription drugs (for nonmedical purposes), other illegal substances, and injected any drugs. Responses for any recent use were recoded and dichotomized for each substance (Yes/No). We describe the sample and characterize recent substance use by using univariate statistics. Logistic regression was used to identify associations between BIPOC status, TGE status, and HIV status and each substance use behavior. We built logistic regression models for each substance use outcome, adjusted for age, sexual orientation, and housing status. Analyses were performed using Stata (version 17; StataCorp LLC).

### Ethical Considerations

The study protocol was approved by the University of California, San Francisco institutional review board (20-33169). Participants provided signed informed consent and were remunerated US $30 (gift card) for completing the assessment.

## Results

The sample was racially/ethnically diverse (Table 1), with 58.68% (240/409) of the sample comprised of BIPOC participants. One in 5 participants (82/409, 20.05%) were TGE, and nearly a third (131/409, 32.03%) were people living with HIV. Marijuana (220/409, 53.79%) and other illegal substance use (227/409, 55.50%) were the most reported, followed by binge drinking (160/409, 39.12%), tobacco use (155/409, 37.90%), and vaping (121/409, 29.58%). Prescription and nonprescription opioid use (such as heroin or fentanyl) was reported by a small proportion of the sample (6/409, 1.47% and 11/409, 2.69%, respectively), while other prescription drug use was 11.74% (48/409), and 10.51% (43/409) reported injection drug use. BIPOC participants had higher odds of tobacco use compared to White participants (adjusted odds ratio [aOR]=1.77, 95% CI 1.13-2.80). TGE participants also had significantly greater odds of tobacco use compared to cisgender men (aOR=3.18, 95% CI 1.70-6.12). People living with HIV had significantly higher odds of both tobacco use (aOR=1.78, 95% CI 1.10-2.89) and injection drug use (aOR=3.14, 95% CI 1.53-6.69) compared to counterparts not living with HIV (Table 2).

**Table 1:** Demographic characteristics and substance use behaviors among online help-seeking sexual and gender minoritized people in San Francisco, California, in 2021-2024 (N=409).

				Values, n (%)
**Demographics**				
	**Age (y)**			
		18-29	103 (25.18)	
		30-39	111 (27.14)	
		40-49	77 (18.83)	
		50+	118 (28.85)	
	**Race/ethnicity**			
		White	169 (41.32)	
		Latino/a/x/e	113 (27.63)	
		Asian, Pacific Islander, and Native Hawaiian	50 (12.22)	
		Black	38 (9.29)	
		Multiracial/other	39 (9.54)	
	**Gender**			
		Cisgender man	327 (79.95)	
		Transgender women or gender expansive	82 (20.05)	
	**Sexual orientation**			
		Bisexual	47 (11.49)	
		Gay/lesbian	282 (68.95)	
		Other	48 (11.74)	
		Straight/heterosexual	32 (7.82)	
	**HIV status**			
		Person not living with HIV	278 (67.97)	
		Person living with HIV	131 (32.03)	
	**Housing stability**			
		Stable	337 (82.40)	
		Unstable	72 (17.60)	
	**Socioeconomic status**			
		Above Federal Poverty Line	337 (82.40)	
		Below Federal Poverty Line	72 (17.60)	
**Substance use behaviors (past 30 days)**				
	**Tobacco**			
		No	254 (62.10)	
		Yes	155 (39.90)	
	**Vaping**			
		No	288 (70.42)	
		Yes	121 (29.58)	
	**Binge drinking**			
		No	249 (60.88)	
		Yes	160 (39.12)	
	**Marijuana**			
		No	189 (46.21)	
		Yes	220 (53.79)	
	**Prescription opioids**			
		No	403 (98.53)	
		Yes	6 (1.47)	
	**Nonprescription opioids**			
		No	398 (97.31)	
		Yes	11 (2.69)	
	**Other prescription drugs**			
		No	361 (88.26)	
		Yes	48 (11.74)	
	**Other illegal substances**			
		No	182 (44.50)	
		Yes	227 (55.50)	
	**Injection drug use**			
		No	366 (89.49)	
		Yes	43 (10.51)	

**Table 2:** Associations between substance use and social inequity among online help-seeking sexual and gender minoritized people in San Francisco, California, 2021-2024 (N=409).

	BIPOC^a^, aOR^b^ (95% CI)	TGE^c^, aOR (95% CI)	PLWH^d^, aOR (95% CI)
Tobacco	1.77 (1.13-2.80)	3.18 (1.70-6.12)	1.78 (1.10-2.89)
Vaping	0.98 (0.61-1.58)	1.05 (0.54-2.00)	1.16 (0.68-1.98)
Binge drinking	0.74 (0.48-1.15)	0.67 (0.34-1.30)	0.57 (0.35-0.93)
Marijuana	1.10 (0.72-1.67)	1.34 (0.73-2.52)	0.92 (0.59-1.46)
Illicit opioids	2.82 (0.68-19.20)	4.14 (0.94-17.30)	0.52 (0.07-2.32)
Prescription opioids	0.68 (0.11-5.60)	0.67 (0.06-5.53)	0.47 (0.05-3.21)
Other prescription drugs	1.03 (0.55-1.98)	0.85 (0.31-2.11)	0.95 (0.46-1.89)
Other illegal drugs	1.10 (0.72-1.69)	0.59 (0.31-1.11)	1.43 (0.89-2.30)
Injection drug use	1.03 (0.51-2.11)	1.15 (0.39-3.17)	3.14 (1.53-6.69)

^a^BIPOC: Black, indigenous, and people of color.

^b^aOR: adjusted odds ratio.

^c^TGE: transgender women and gender expansive.

^d^PLWH: people living with HIV.

## Discussion

Our findings characterize the diverse substance use behavior profiles of SGM people seeking help online in San Francisco. Tobacco use showed a disparity across all groups, likely due to exposure to social stress and discrimination [9]. We also found that people living with HIV had significantly greater odds of injection drug use—a finding that may reflect the syndemic relationship between substance use and HIV as well as persistent structural vulnerabilities. The generalizability of this study is limited by its use of online recruitment and focus on San Francisco, a location that has successfully adopted harm reduction policies [10]. Despite this, community-based studies can supplement nationally representative, publicly available datasets that may undersample SGM and BIPOC communities. While much of substance use research has examined illicit substance use among SGM communities, our findings are a sober reminder that the need for tobacco cessation is also great. The implications of these findings may include bolstering local public health efforts to utilize digital methods to conduct outreach and deliver brief behavioral interventions directly to communities actively seeking help.

## Abbreviations

aOR: adjusted odds ratio
BIPOC: Black, indigenous, and people of color
SGM: sexual and gender minoritized
TGE: transgender women and gender expansive

## Data Availability

The datasets generated or analyzed during this study are not publicly available as they contain details that could be used to identify participants but are available from the corresponding author on reasonable request.
